# Testing the aminostratigraphy of fluvial archives: the evidence from intra-crystalline proteins within freshwater shells

**DOI:** 10.1016/j.quascirev.2007.06.034

**Published:** 2007-11

**Authors:** K.E.H. Penkman, R.C. Preece, D.H. Keen, D. Maddy, D.C. Schreve, M.J. Collins

**Affiliations:** a BioArch, Departments of Biology, Archaeology and Chemistry, Biology S Block, University of York, P.O. Box 373, York YO10 5YW, UK; b Department of Zoology, University of Cambridge, Downing Street, Cambridge CB2 3EJ, UK; c Institute of Archaeology and Antiquity, University of Birmingham, Birmingham B15 2TT, UK; d School of Geography, Politics and Sociology, Daysh Building, Newcastle University, Newcastle-upon-Tyne NE1 7RU, UK; e Department of Geography, Royal Holloway, University of London, Egham, Surrey TW20 0EX, UK

## Abstract

Until recently few studies of amino acid racemization of fossil bivalves and gastropods collected from river terrace deposits in Europe were based on the analysis of the intra-crystalline fraction. Instead they were based on the epimerization (racemization) of a single amino acid, isoleucine, and its inter-conversion to alloisoleucine. This paper presents data from the analysis of the intra-crystalline fraction of the shells, using a preparation technique of sample bleaching to remove the leachable matrix, thus leaving a component that exhibits closed-system behaviour. Reverse-phase HPLC separation with fluorescence detection allows the interpretation of four amino acids in detail: aspartic acid, glutamic acid, alanine and valine. The intra-crystalline fraction offers greater potential for improved resolution, especially when combined with the analysis of multiple amino acid d/l values, which racemize at different rates. This is explored using three species of freshwater gastropods (*Bithynia tentaculata and troschelii*, *Valvata piscinalis*) and the bivalve *Corbicula*. Sites of different ages within the Lower Thames river terrace sequence are used as a stratigraphical framework, with samples from other southern UK sites providing supplementary evidence. The results indicate better resolution using the intra-crystalline fraction over that obtained using unbleached shells, with differentiation possible at sites of up to MIS 7 age. However, for older sites, although values are always higher, the separation is less successful. A species effect has been identified between the gastropod shells. Despite the analysis of intra-crystalline protein, amino acid data from *Corbicula* remain problematical. Preliminary data on the opercula from *Bithynia* indicate that better resolution is possible, particularly at older sites.

## 1 Introduction

Across western Europe many large-scale river terrace sequences preserve sedimentary archives that appear to reflect fluvial system responses driven by climate change at orbital (Milankovitch) frequencies (Bridgland et al., 2004; Bridgland 2006; Bridgland and Westaway, 2007), but the precise timing of events requires accurate dating of the deposits. Such geochronological age estimates are provided by various means: radiocarbon, U-series, electron spin resonance (ESR), optically stimulated luminescence (OSL) and amino acid geochronology, allowing correlation with stages of the oxygen isotope framework of the global ocean, which in turn is correlated with variability in the orbital parameters of obliquity and precession (Imbrie et al., 1984).

Amino acid racemization (AAR) depends on the slow inter-conversion (racemization) of l-amino acids, the basic building blocks of protein, into an equilibrium mixture of l- and d-amino acids in fossils over time. Even in an optimal set of samples, with closed system behaviour and identical (internal) chemical environments for degradation, aminostratigraphic correlation between sites relies on the samples sharing an equivalent temperature history (e.g. Wehmiller et al., 2000). Bowen et al. (1989) provided the first comprehensive aminostratigraphic framework of non-marine deposits in southern Britain, based upon the extent of a single AAR reaction (A/I epimerization) in mollusc shells, proposing correlations of the deposits with the marine oxygen isotope record. This framework has been refined with the addition of further analyses and the inclusion of further sites (e.g. Bowen, 1999, 2000) and modified most notably in the case of Hoxne (Bowen, 2003) as the result of new ESR age estimates (Grün and Schwarcz, 2000).

This paper presents the results of improvements in sample pre-treatment and new analytical methods (Penkman, 2005; Penkman et al., 2007). Instead of using only isoleucine, as in previous work, multiple amino acids are analysed from the intra-crystalline fraction of *Valvata*, *Bithynia* and *Corbicula* shells. For the period under scrutiny, the last ∼450 ka, it confirms that there is a relationship between the age of terraces in the Lower Thames and the extent of racemization.

## 2 Materials and methods

### 2.1 Materials

Rates of racemization may vary significantly between species (Miller and Hare, 1975; Lajoie et al., 1980). For marine taxa, amino acid ratios of fast, moderate and slow racemizers (Miller and Mangerud, 1985) are often standardized to a common reference species (e.g. Bowen et al., 1985; Bowen and Sykes, 1988). Racemization rates also vary between non-marine taxa (Hughes, 1987) and although they were standardized for the study of the Somme and Seine terraces (Bates, 1993), it was thought that insufficient data were available for a study across different environments in southern England and assumed that rates of A/I epimerization were approximately the same (Bowen et al., 1989). In this study we test this assumption on the intra-crystalline fraction by investigating four taxa abundant in fluvial sediments throughout Europe and commonly used for aminostratigraphy. Three of these are prosobranch gastropods: *Valvata piscinalis* (Müller), which in NW Europe occurs commonly in both temperate and cold stage sediments, and *Bithynia tentaculata* (L.) and *B. troschelii*, which are known only from interglacial or interstadial deposits. The fourth species is the bivalve *Corbicula fluminalis* (Müller), which in Britain is known only from interglacial sediments but not from those of Last Interglacial age (Keen, 1990; Meijer and Preece, 2000). The systematics of *Corbicula* are complex and attribution of the Pleistocene shells to *C. fluminalis* is provisional (Meijer and Preece, 2000). An earlier Tertiary species, *Corbicula obovata* (J. Sowerby) was also analysed to test whether AAR values were increased in samples significantly older than the Pleistocene material in question. Previous studies have shown that ratios from *Corbicula* vary systematically, depending on whether the samples were taken from the umbonal region or the outer margin (West et al., 1999); all samples in this study have been taken from the outer margin. *V. piscinalis*, *B. tentaculata*, *C. fluminalis* and *C. obovata* shells have therefore been analysed from a range of sites, detailed in the Online Supplement.

### 2.2 Methods

All samples were prepared using the procedures of Penkman (2005) and Penkman et al. (2007). In brief, each sample was powdered and bleached for 48 h with 12% NaOCl to isolate the intra-crystalline fraction. Bleaching removes unwanted (contaminating or partly leached) proteins from gastropod samples (Penkman et al., 2007), reducing within-site variability. Some samples were also analysed unbleached, to enable comparison with previous methods. Two subsamples were then taken: one fraction was directly demineralized and the free amino acids analysed (referred to as the ‘free amino acid’ fraction; FAA; ‘F’), and the second was treated with 7 M HCl under N_2_ at 110 °C for 6 h or 24 h to release the peptide-bound amino acids, thus yielding the ‘total hydrolysable amino acid’ concentration (THAA; ‘H’). The shorter hydrolysis time (6 h) did not appear to break all the peptide bonds; therefore, in subsequent experiments, 24 h hydrolysis was used. Samples were then dried and rehydrated for Reverse Phase High Pressure Liquid Chromatography (RP-HPLC) analysis with l-homo-arginine as an internal standard.

The amino acid compositions of the samples were analysed in duplicate by RP-HPLC using fluorescence detection, following a modified version of the method of Kaufman and Manley (1998). A sample is injected and mixed online with the derivitising reagent (*n*-iso-l-butyryl l-cysteine (IBLC) and *o*-phthaldialdehyde (OPA)). The amino acids are separated on a C_18_ HyperSil BDS column at 25 °C using a gradient elution of three solvents: sodium acetate buffer, methanol and acetonitrile. During preparative hydrolysis both asparagine and glutamine undergo rapid irreversible deamination to aspartic acid and glutamic acid, respectively (Hill, 1965). It is therefore not possible to distinguish between the acidic amino acids and their derivatives and they are reported together as Asx and Glx. Error bars for the duplicate analyses are not included in the figures, for the sake of clarity, but an alternative set of figures, with error bars, is included in the Online Supplement. Although some samples analysed would not pass the criteria for closed system protein (Preece and Penkman, 2005), all data obtained is shown. Statistical analysis was performed using Minitab v.14.

### 2.3 Pre-treatment developed to isolate a closed system

In order to overcome problems arising from burial diagenesis, it is argued that the protein system must remain closed from synthesis to analysis (Brooks et al., 1990; Collins and Riley, 2000; Miller et al., 2000). In a closed system, diagenetic reactions of indigenous biomolecules should be predictable. Consequently the distribution between original molecules and their degradation products can theoretically be used to interpret the diagenetic history and to identify samples contaminated by exogenous amino acids and/or those that have lost part of their original protein. A closed system has the further advantage of a consistent chemical environment, including narrow pH range, as well as consistent ion and water concentrations (Towe, 1980; Sykes et al., 1995). The success of avian eggshell as a material for amino acid geochronology has been attributed to its approximation to a closed system (Brooks et al., 1990; Miller et al., 2000). Mollusc shell, more widely available as dating material, does not form a completely closed system (Miller and Hare, 1980; Roof, 1997), retaining only 60% of its original protein in experimental studies. However, a component of the protein within mollusc shells does appear to be effectively ‘closed’ and immune from environmental effects. This ‘intra-crystalline’ fraction can be isolated by bleaching, removing the inter-crystalline matrix and any exogenous amino acids (Towe, 1980). Preliminary results of bleaching in terrestrial gastropods have provided improved datasets (Sykes et al., 1995), as have results from a larger investigation on both modern Holocene aquatic shells and their Pleistocene counterparts (Penkman et al., 2007). Indeed the latter study demonstrated for the first time that the intra-crystalline fraction behaved as a closed system, both experimentally and in fossil shells. Bleach pre-treatment removes any partly degraded/leached and contaminating proteins, providing a closed-system protein fraction with more tractable kinetics. As a consequence of the ability to isolate a closed system in gastropod shells, it has been possible to measure routinely not only the THAA (all the amino acids present in the system, the conventional fraction for analysis), but also to obtain reliable data from the FAA fraction (those amino acids that are not peptide-bound). Only in a well-behaved shell will there be a consistent relationship between the data from these two fractions, as the FAA fraction is much more readily lost from the shell than the peptide-bound amino acids. This approach is useful as it is possible to sample different fractions of protein diagenesis from within the same sample, therefore obviating the need for extensive screening of the data based on THAA values only (Bowen, 2000).

### 2.4 Advantages of analysing multiple amino acids

Previous work only utilised the extent of racemization (epimerization) of one amino acid, l-isoleucine to d-alloisoleucine, although some 12 other amino acids were resolved and used as a check on HPLC chromatograms (Bowen, 2000). However, a further refinement used here involves a new chromatographic technique, which uses reverse-phase separation with fluorescence detection. This now permits both the discrimination of multiple chiral amino acids and their detection at low concentrations from smaller sample sizes (Kaufman and Manley, 1998). The advantages offered by this technique are twofold. Firstly, the number of degradation reactions that can be used to interpret the diagenetic history is increased, so that fast-racemizing amino acids can be used on shorter timescales and slower ones on longer timescales. Secondly, although the reaction rates are different, they should be correlated, providing a check on the integrity of the samples (Preece and Penkman, 2005). The l and d isomers of 10 amino acids were routinely detected, but the amino acids studied in detail were those whose both d and l enantiomers were well resolved: Asx, Glx, serine (Ser), alanine (Ala) and valine (Val).

### 2.5 Testing the techniques: the terrace sequence of the Thames valley

The amino acid results obtained in this study need to be tested against a secure stratigraphical template. The terrace sequence of the Thames valley is arguably one of the best studied in Europe and provides the longest and potentially most-complete sequence of Pleistocene sediments in Britain. Opinions differ about the attribution of certain sites in the Thames valley to specific stages, and especially how they correlate with the marine oxygen isotope record (Gibbard, 1985, 1994; Bridgland, 1994), but there is no doubt from their location in the terrace staircase about their relative stratigraphical positions. All the Thames sites analysed are younger than the Anglian Stage, normally attributed to Marine Oxygen Isotope Stage (MIS) 12 (Bowen, 1999) during which the Thames was glacially diverted into its present valley. In the Middle and Lower Thames four terraces contain interglacial deposits that are thought to have accumulated during MIS 11, 9, 7 and 5e (Bridgland, 1994). This model is supported by several lines of evidence, including the original amino acid A/I data, molluscan and mammalian biostratigraphy (e.g. Bowen et al., 1989, 1995; Preece, 1995; Schreve, 2001). This sequence provides a valuable stratigraphical template against which to gauge the new amino acid data. As an additional check on the veracity of the data from the Thames, new amino acid analyses have also been undertaken on sites outside of the Thames valley that have good independent evidence of age, some of which also contain important Palaeolithic archaeology. Although the majority of sites are fluvial, lacustrine sites are also included, as this technique has the potential to correlate a range of freshwater sediments. Critical evidence of their age is given in the Online Supplement.

**Fig. 1:**
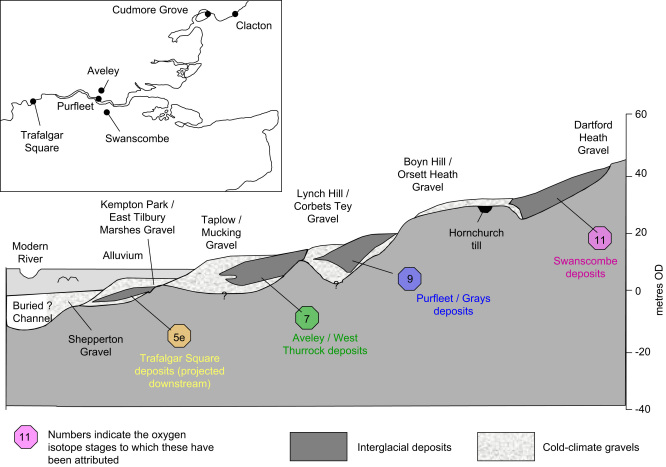
Lower Thames terrace staircase (after Bridgland, 1994), with inset showing location of Thames sites.

The locations of the Lower Thames sites under test are given in Fig. 1, with details as follows:

*Swanscombe*: Dierden's Pit (Ingress Vale; TQ 595748) is part of the complex of sites near Swanscombe. The shelly gravels have yielded the so-called ‘Rhenish fauna’ including *Theodoxus danubialis*, *Viviparus diluvianus* and *Valvata naticina* (Kerney, 1971; see also Schreve et al., 2007), which allows correlation with the Middle Gravels of the nearby Barnfield Pit sequence (Grid ref: TQ 595745), the horizon containing the celebrated hominin skull (Ovey, 1964; Kerney, 1971). The geomorphological and stratigraphical positions of these gravels within the Orsett Heath Formation (Bridgland, 1994) show that they post-date the Anglian glaciation (Gibbard, 1985; Bridgland, 1994). A/I amino acid geochronology, molluscan and mammalian biostratigraphy support a Hoxnian (MIS 11) age (Kerney, 1971; Turner and Kerney, 1971; Bowen et al., 1989, 1995; Bridgland, 1994; Preece, 1995; Schreve, 2001).

*Purfleet*: Two quarries on the northern bank of the Thames at Purfleet (Bluelands Pit, TQ 560784, and Greenlands Pit, TQ 568785) contain highly fossiliferous gravel, sand and intertidal silt deposits of up to 5 m in thickness (Hollin, 1977). The Purfleet Member is recognised as an independent unit, within the Corbets Tey Gravel Formation, by Bridgland (1994), with tentative correlation to MIS 9 indicated by the A/I amino acid geochronology, lithostratigraphy, terrace stratigraphy and biostratigraphy (Bowen et al., 1995; Bowen, 2000; Schreve et al., 2002).

*Aveley/Lion Pit Tramway Cutting*: The Aveley Member stratotype is at Sandy Lane Quarry (TQ 552808), now infilled, where the fossiliferous sediments consist of gravel, sand, silt and detritus mud, overlying bedrock or earlier Pleistocene sediments (Hollin, 1977; Gibbard, 1994; Bridgland, 1994). The deposits at Aveley were originally correlated with the Ipswichian (West, 1969), but their elevation above that of the Trafalgar Square sediments, together with different mammalian assemblages, led Sutcliffe (1964, 1975) and Sutcliffe and Bowen (1973) to suggest that they were older than the Ipswichian. The terrace succession model of Bridgland (1994; but see Gibbard, 1994 for an alternative view) places the interglacial sediments within the Mucking Formation, the third of four terraces of the Lower Thames thought to post-date the Anglian. The Lion Pit tramway cutting at West Thurrock, Essex (TQ 598783) exposes fluvial gravel and sand up to 9 m in thickness (Schreve et al., 2006) that overlie bedrock at 4 m O.D. (Gibbard, 1994). This site also lies in the Mucking Formation of the Lower Thames (Bridgland, 1994). Support for a correlation with MIS 7 for both sites has come from A/I amino acid geochronology (Bowen et al., 1989) and biostratigraphy based on molluscs (Keen, 1990) and mammals (Schreve, 2001).

*Trafalgar Square*: The stratotype of the Trafalgar Square Member is at Canada House, Trafalgar Square, London (TQ 292805) (Bowen, 1999). The thick interglacial deposits, up to 12 m of gravel, sand, silt and detritus mud, are part of the Kempton Park Formation (Bridgland, 1994, 2006), formerly the ‘Upper Floodplain Terrace’ of the Thames. The highly fossiliferous deposits vary in nature from shelly sands to organic muds and are correlated by means of biostratigraphy (Franks, 1960; Sutcliffe and Bowen, 1973; Gibbard, 1985; Preece, 1999) and aminostratigraphy (Bowen et al., 1989; Bridgland, 1994) with the Ipswichian and therefore with MIS 5e (Bowen et al., 1989; Bowen, 1999).

## 3 Results

**Fig. 2:**
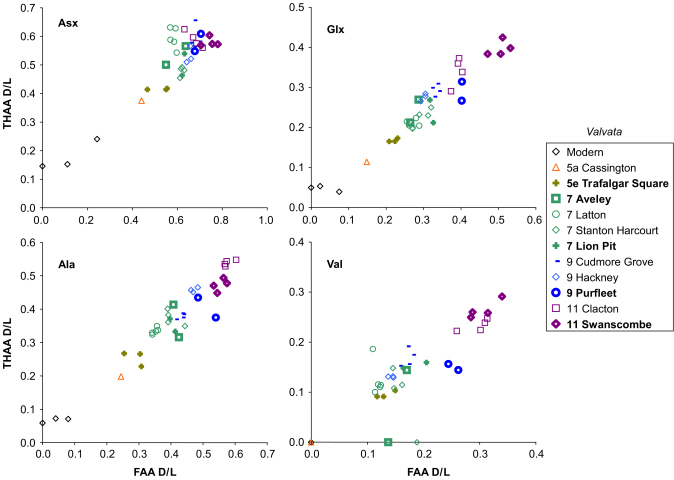
THAA vs. FAA for Asx, Glx, Ala and Val d/l in the bleached (intra-crystalline) fraction of *Valvata piscinalis* from the Lower Thames terrace sequence and other sites in the Thames/Medway system.

**Fig. 3:**
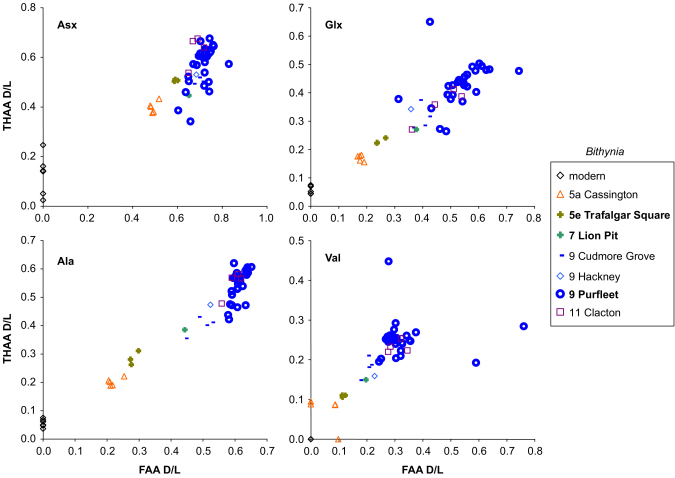
THAA vs. FAA for Asx, Glx, Ala and Val d/l in the bleached (intra-crystalline) fraction of *Bithynia tentaculata* shells from the Lower Thames terrace sequence and other sites in the Thames/Medway system. Although some samples fail the closed system test of high correlation between FAA and THAA, all data are shown; note the large spread in data from Purfleet.

**Fig. 4:**
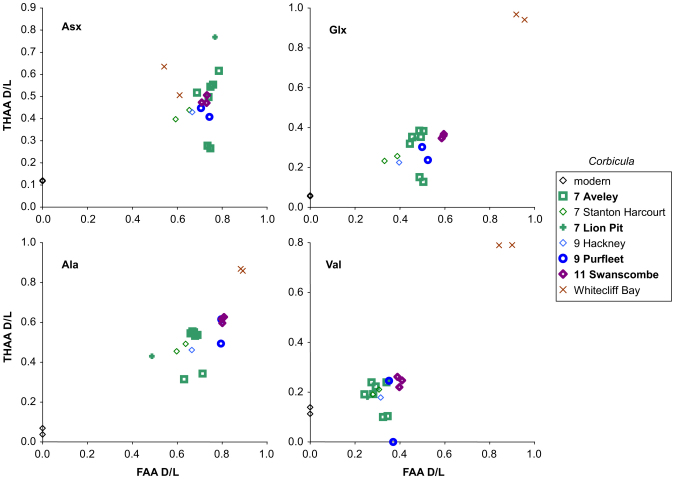
THAA vs. FAA for Asx, Glx, Ala and Val d/l in the bleached (intra-crystalline) fraction of *Corbicula* sp. from the Pleistocene Thames terrace sequence, other sites in the Thames/Medway system and the Oligocene/Eocene Bembridge Marls at Whitecliff Bay, Isle of Wight.

The two separate analyses made on each individual sample, the FAA and THAA, should show a strong positive correlation for each amino acid d/l. This is because the extent of racemization in the THAA is, in large part, driven by peptide-bond hydrolysis, which in turn liberates FAA (Miller et al., 2000). A lack of the expected correlation between FAA and THAA will reveal samples in which the closed system may have been compromised (e.g. Preece and Penkman, 2005). Plotting the extent of racemization of THAA against FAA presents the samples in relative aminostratigraphical order based on their d/l's, with more degraded samples having higher d/l values for both the FAA and THAA fractions (e.g. Figs. 2–4). Occasionally concentrations were too low for detection in one fraction; in those cases the data from one fraction plot along the zero-axes.

### 3.1 Species effect

The results from *Corbicula* are discussed in more detail below but, within the gastropod shells, differences were found in the absolute d/l values within the intra-crystalline fraction of *Bithynia* and *Valvata* samples from the same site (Online Supplement Fig. 1). Along with the problem of small sample sizes, amino acid data has upper and lower limits (0 and 1) and therefore statistical tests must be applied with caution. However, tests for normality were undertaken on each dataset and two types of statistical tests applied in order to provide a useful insight alongside the interpretation based on the graphical data alone. For each amino acid within each fraction, 2-tailed *t*-tests (for normally distributed data) and Mann–Whitney tests (for non-normal data) were undertaken on the data from four Thames sites: 14/32 2-tailed *t*-tests and 10/32 Mann–Whitney tests showed a significant difference (Online Supplement Worksheet 4; the MIS 9 site at Cudmore Grove was substituted for Purfleet due to the diagenetic problems at Purfleet discussed below). However, the differences do not remain consistent as the samples increase in age and although the data from *Bithynia* is usually slightly higher, this is not true in all cases. The intra-crystalline proteins extracted from the two different gastropod species are sufficiently different to alter the rates of protein breakdown and hence observed d/l. It is therefore necessary to develop aminostratigraphic frameworks for each individual species.

### 3.2 AAR from Thames sites

Figs. 2–4 show the THAA vs. FAA for the intra-crystalline (bleached) data for Asx, Glx, Ala and Val for the Lower Thames sites of Trafalgar Square, Aveley/Lion Pit, Purfleet and Swanscombe. As a further check, data is presented from additional sites in the Thames system (Cassington, Latton, Stanton Harcourt, Cudmore Grove, Hackney and Clacton). For each of the amino acids, the modern samples lie at the lowest FAA and THAA values. The strong positive correlation observed in the bleached gastropod THAA vs. FAA graphs (Figs. 2–3) indicate that the extent of protein degradation in their shells is an indication of the relative age of the sites.

Within a closed system AAR is driven by the extent of protein degradation, itself a function of the time/temperature history of a sample. Less degradation occurs during cold stages and as a consequence we have used statistical tests on the *V. piscinalis* shells to establish whether it is possible to use the extent of AAR to discriminate between interglacial shells found within different terrace levels from the Lower Thames terrace staircase. For each amino acid within each fraction (FAA and THAA), both 1-tailed and 2-tailed *t*-tests and Mann–Whitney tests were performed on the Trafalgar Square, Aveley/Lion Pit, Purfleet and Swanscombe data. If the result of the *t*-test produces a *p*<0.05, this enables discrimination between the two sites at a 95% confidence level. The 1-tailed *t*-test assumes prior knowledge of the stratigraphy of the sites whereas the 2-tailed *t*-test assumes no prior knowledge and therefore provides a more useful guide of the resolving power of this technique when applied to sites of unknown age. The full results are presented in the Online Supplement but are also summarized in Table 1: in general, discrimination is better for the younger sites. The data tend to support the model of Bridgland (1994), with deposits present in the Lower Thames sequence intermediate in age between the Ipswichian (MIS 5e) and the Hoxnian (MIS 11), although the level of resolution in the older material (>MIS 7) allows this to be stated with less certainty. The results reveal that there is sufficient coherence within a single dataset to enable statistical discrimination between sites, something that McCarroll (2002) had urged racemization researchers to address.

*Corbicula* has previously been identified as yielding problematic amino acid data at some sites, notably at Purfleet (Miller et al., 1979; Bowen et al., 1995), although unbleached shells have provided A/I values consistent with other species at other sites (Miller et al., 1979; Hughes, 1987; Bowen et al., 1989). However, bleaching did not appear to improve the data obtained from this bivalve in fossil samples (Penkman et al., 2007). As can be seen (Fig. 4), the d/l values of none of the amino acids selected enable discrimination between the MIS 7 and MIS 9 sites. The samples from Aveley show a large scatter, but the d/l values from this site generally exhibit some of the highest values, despite the likelihood that it is younger than both Hackney and Purfleet. However, high d/l values consistent with a Tertiary age were obtained from *C. obovata* from the Bembridge Marls at Whitecliff Bay, Isle of Wight (see Gale et al., 2006 for discussion of age).

#### 3.2.1 Purfleet

In contrast to other sites studied in the Thames Valley, which have yielded A/I results on whole shell consistent with their relative stratigraphic context, the sites at Purfleet have previously given amino acid ratios seriously overestimating their age (Miller et al., 1979; Bowen et al., 1989) although subsequent analyses from *Valvata* were consistent with the stratigraphical position of the site (Bowen et al., 1995). The data from the intra-crystalline fraction of *Bithynia* and *Valvata* show a large scatter (Figs. 2 and 3). Carbonate concretions (‘race’) occur at the site, formed not only around stones, but also around freshwater mussels, leaving the internal casts of the shells (Schreve et al., 2002). This clearly demonstrates that bicarbonate has been leached and re-deposited. Such post-depositional diagenetic processes are likely to be implicated in the anomalous amino acid results obtained.

### 3.3 AAR from all sites

**Fig. 5:**
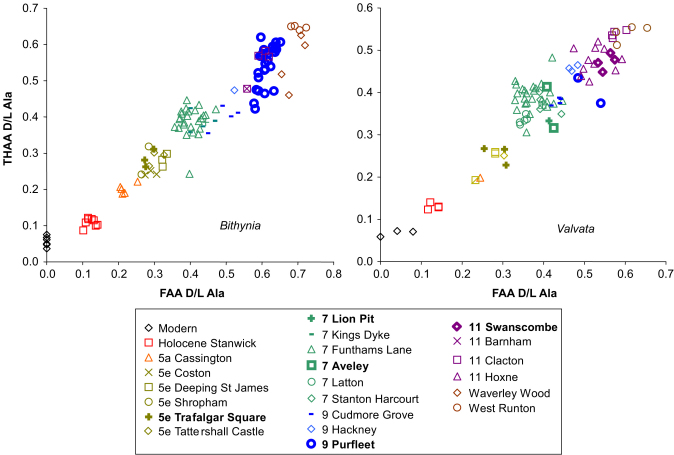
THAA vs. FAA for Ala d/l in the bleached (intra-crystalline) fraction from shells of *Bithynia tentaculata* and *B. troschelii* (left) and *Valvata piscinalis* (right). The colouring represents the independent age evidence for each site: black=modern, red=Holocene, orange=MIS 4/5a, yellow=Ipswichian/MIS 5e, green=MIS 7, blue=MIS 9, purple=Hoxnian/MIS 11, brown=pre-MIS 12 ‘Cromerian Complex’.

Further data from other sites in southern England are included in Fig. 5 and provide a wider context. The points from each site have been coloured according to independent geochronological age estimates that correlate them with oxygen isotope stratigraphy (see Online Supplement for details; for all four of the amino acids and the unbleached dataset see Online Supplement Figs. 2–6). As previously reported, bleaching reduces the variation within a single site compared to unbleached samples (Online Supplement Figs. 2–6; for example note the decrease in scatter in the Hoxne dataset upon bleaching, Online Supplement Fig. 4), simplifying interpretation of the data. It is therefore this bleached intra-crystalline dataset that is interpreted in detail here.

There is an increase in the extent of racemization with time in the bleached gastropod shells (Figs. 2, 3 and 5). As expected, there has been little racemization within cold stages, as the reaction rates of protein breakdown would have slowed at these lower temperatures. In samples correlated with part of MIS 7 age or younger, the extent of racemization is sufficient to differentiate the sites already separated by lithostratigraphy and geomorphology (Bridgland, 1994). MIS 7 is fairly well differentiated from later stages in some amino acids. In the case of Asx, which undergoes rapid racemization, there is a clustering of data beyond MIS 5, but in slower racemizing amino acids such as Glx, Ala and Val, discrimination is better. Sites older than MIS 7 show higher levels of racemization, but separation is increasingly less clear with age (Fig. 5; Online Supplement Figs. 2–3).

## 4 Discussion

Debate over the existence of two or more interglacials in the UK record since the Anglian (MIS 12) is of longstanding (Bowen, 1978; Shotton et al., 1983) and research has focused on the Thames fluvial deposits in an attempt to solve this question. Gibbard (1985, 1994) proposed that only two interglacials were represented in the Thames terraces after the Anglian diversion: the Hoxnian (now correlated with MIS 11), such as at the site at Swanscombe, and the Ipswichian (correlated with MIS 5e) represented by the Trafalgar Square deposits. Sediments at intermediate elevations, such as those at Purfleet and Aveley, were also attributed to the Ipswichian by Gibbard (1994), their additional height explained as due either to deposition by Thames tributaries or to sea-level rise at this time. Bridgland (1994), on the other hand, identified four interglacials in the same post-diversion sequence, as did the A/I aminostratigraphical model of Bowen et al. (1989).

The applicability of the new AAR technique reported here also shows that the amino acid data are consistent with the stratigraphical position of the terraces and support the model of four post-Anglian interglacials. Good separation is observed between the younger sites and it may be possible to separate events at the Oxygen Isotope sub-stage level up to and including MIS 7. Older events are not separated with such clarity but the method does not fail completely, with increasingly higher racemization values in the slower racemizing amino acids.

Interspecies variation, as reported in other studies of whole shell (e.g. Lajoie et al., 1980), was also observed in the intra-crystalline fraction. Somewhat counter-intuitively, small, relatively thin-shelled gastropods yield more consistent data than the much more robust shells of the bivalve *Corbicula*, although we suspect that this is due to the different protein compositions of the different ultra-structural layers in this species (Penkman et al., 2007).

The different amino acids that contribute to the entrapped protein within the biomineral undergo racemization at different rates, reflecting differences in the mechanisms of isomerization and the varying stability of their respective peptide bonds. Consequently, by analysing multiple amino acid dl ratios, the overall range of discrimination between terraces is extended. By combining these different d/l values, it is possible to assess the overall state of protein degradation with a greater degree of confidence. This technique has the advantage in that it draws information from multiple amino acids and also from the extent of racemization in both FAA and THAA, therefore providing multiple isochrons. Unusual within-amino acid patterns are identifiable, because although racemization rates differ between individual amino acids, they should be highly correlated in a closed system and therefore examination of d/l patterns within a single sample provides an element of quality control.

**Fig. 6:**
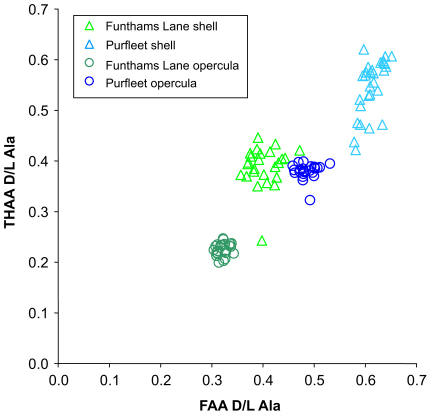
Left: THAA vs. FAA d/l Ala for bleached *Bithynia* shells and opercula from Funthams Lane and Purfleet. Each of the measurements is from a single shell or operculum. Note the variation in relative rates between the two biominerals and the much greater consistency in the measurements obtained from opercula. The outlying shell sample has an amino acid concentration two times that of the other shells, indicating the importance of adequate bleaching (Penkman et al., 2007).

Despite the advantages of the simple bleaching preparation step to isolate intra-crystalline amino acids, it is in itself insufficient to yield data from gastropod shells able to clearly resolve sites older than MIS 7, due to increased levels of within-site variability. Recently the ability to discriminate events within the ‘Cromerian Complex’ using a different biomineral, the calcitic operculum from one of the gastropods studied here, *Bithynia* sp., has been presented (Penkman et al., accepted). The striking difference in resolution between gastropod shells, which are made of aragonite, and the opercula, suggests that the diagenetic transformation of aragonite may be partially responsible for the poor resolution observed in older gastropod samples; higher concentrations of intra-crystalline amino acids within the opercula may also contribute to improved discrimination. A previous study has shown that isoleucine epimerization measurements on unbleached *Bithynia* opercula from five sites, including Trafalgar Square and Aveley, also yielded lower and more consistent ratios (Hughes, 1987). The level of natural variability within a horizon has significant impacts on the level of resolution that can be obtained from the amino acid data and the low levels of variability observed within the bleached opercula in both Cromerian (Penkman et al., accepted) and younger material, including that from the problematic site at Purfleet (Fig. 6) indicate that analysis of this biomineral has the potential to increase the level of resolution of this technique.

## 5 Conclusions

Using the new technique of intra-crystalline amino acids analysed by RP-HPLC, it is shown that the extent of degradation in skeletal protein from freshwater gastropods makes an important contribution to the interpretation of fluvial deposits. The organic matter entrapped within the biomineral (intra-crystalline fraction) is believed to be a more reliable fraction for analysis than the whole shell (Sykes et al., 1995; Penkman et al., 2007), with the preparatory bleaching step removing both secondary contamination and any residual (inter-crystalline) organic matrix, which can degrade and leach at an unpredictable rate over time. Analysis of multiple amino acids in both the FAA and THAA fractions shows an increase in the extent of protein degradation with time in commonly occurring gastropod shells, enabling correlations to be made between both fluvial and other freshwater sites using AAR. The racemization data reported is different from earlier work that examined d/l values from the THAA of whole mollusc shells containing both intra- and inter-crystalline material. However, the same separation of the Lower Thames terraces has emerged.

The levels of AAR in gastropod shells allow distinction between sites correlated with MI stages and substages up to and including MIS 7. In gastropod shells from deposits higher within the terrace sequence (i.e. older), the extent of racemization is greater still, but the levels of natural variability are higher. The increase in variability may be due to aragonitic shells undergoing diagenetic alteration to calcite and it is possible that with careful selection of unaltered carbonates, the aminostratigraphic framework for these gastropods could be extended further. Discrimination between sites and the level of natural variability within sites promise to be much better if diagenetically stable calcitic opercula are used. In this study lithostratigraphy and geomorphology of river terraces have provided a stratigraphical framework for the testing of the utility of intra-crystalline AAR for geochronology, but it is apparent that there is value in including AAR as a routine analysis for the study of fluvial records.

## Figures and Tables

**Table 1:** Summary of statistical test results (Minitab), using both 1- and 2-tailed *t*-tests (assuming normal distribution) and Mann–Whitney tests (assuming non-normal distribution)

	Is Trafalgar Square younger than Aveley/Lion Pit?		Is Aveley/Lion Pit younger than Purfleet?		Is Purfleet younger than Swanscombe?	
	Yes	No	Yes	No	Yes	No
*Valvata* (1-tailed *t*-test)	8	0	6	2	7	1
*Valvata* (2-tailed *t*-test)	8	0	5	3	6	2
*Valvata* (1-tailed MW-test)	8	0	5	2	7	1
*Valvata* (2-tailed MW-test)	8	0	4	4	7	1

The results of each duplicate analysis are included in order to provide a statistically significant sample size. Number in “Yes” column represents the number of amino acid (AA) fractions that enable discrimination between the two Thames sites in question, and also supports their stratigraphical position at a 95% confidence level. Number in “No” column represents the number of AA fractions that do not enable discrimination between the two sites. For example: In the 2-tailed *t*-tests, analysis of *Valvata* shell results in eight out of eight AA fractions supporting the hypothesis that Trafalgar Square is distinguishable and younger than Aveley/Lion Pit; only six out of eight support the hypothesis that Swanscombe is older than Purfleet. Full details of the statistical tests are given in the Online Supplement Worksheet 3.

## References

[bib1] Bates M.R. (1993). Quaternary aminostratigraphy in Northwestern France. Quaternary Science Reviews.

[bib2] Bowen, D.Q., 1978. Quaternary Geology: a stratigraphic framework for multidisciplinary work. Pergamon, Oxford, 221pp., Russian translation, Moscow, 272pp., 2nd edition 1985.

[bib3] Bowen, D.Q., 1999. A revised correlation of Quaternary deposits in the British Isles, Geological Society Special Report, No. 23.

[bib4] Bowen D.Q., Goodfriend G.A., Collins M.J., Fogel M.L., Macko S.A., Wehmiller J.F. (2000). Revised aminostratigraphy for land–sea correlations from the northeastern North Atlantic margin. Perspectives in Amino Acid and Protein Geochemistry.

[bib5] Bowen, D.Q., 2003. Uncertainty in Oxygen Isotope Stage 11 sea level: an estimate 13±2 m above low water from Great Britain, In: Droxler, A., Poore, R.Z., Burkle, L.H. (Eds.), Earth's Climate and Orbital Eccentricity: The Marine Isotope Stage 11, Geophysical Monograph 137*,* American Geophysical Union, pp. 131–144.

[bib6] Bowen D.Q., Sykes G.A. (1988). Correlation of marine events and glaciations on the northeast Atlantic margin. Philosophical Transactions of the Royal Society B.

[bib7] Bowen D.Q., Sykes G.A., Reeves A., Miller G.H., Andrews J.A., Brew J.S., Hare P.E. (1985). Amino Acid Geochronology of raised beaches in southwest Britain. Quaternary Science Reviews.

[bib8] Bowen D.Q., Hughes S., Sykes G.A., Miller G.H. (1989). Land–sea correlations in the Pleistocene based on isoleucine epimerization in non-marine mollusks. Nature.

[bib9] Bowen D.Q., Sykes G.A., Maddy D., Bridgland D.R., Lewis S.G., Bridgland D.R., Allen P., Haggart B.A. (1995). Aminostratigraphy and amino acid geochronology of English lowland valleys: the Lower Thames in context. The Quaternary of the Lower Reaches of the Thames, Field Guide.

[bib10] Bridgland, D.R., 1994. Quaternary of the Thames, Geological Conservation Review Series No. 7.

[bib11] Bridgland D.R. (2006). The Middle and Upper Pleistocene sequence in the Lower Thames: a record of Milankovitch climatic fluctuation and early human occupation of southern Britain. Proceedings of the Geologists’ Association.

[bib12] Bridgland, D., Westaway, R., 2007. Climatically controlled river terrace staircases: a worldwide Quaternary phenomenon. Geomorphology, doi:10.1016/j.geomorph.2006.12.032.

[bib13] Bridgland D.R., Maddy D., Bates M. (2004). River terrace sequences: templates for Quaternary geochronology and the marine–terrestrial correlation. Journal of Quaternary Science.

[bib14] Brooks A.S., Hare P.E., Kokis J.E., Miller G.H., Ernst R.D., Wendorf F. (1990). Dating Pleistocene archaeological sites by protein diagenesis in ostrich eggshell. Science.

[bib15] Collins M.J., Riley M.S., Goodfriend G.A., Collins M.J., Fogel M.L., Macko S.A., Wehmiller J.F. (2000). Amino acid racemization in biominerals, the impact of protein degradation and loss. Perspectives in Amino Acid and Protein Geochemistry.

[bib16] Franks J.W. (1960). Interglacial deposits at Trafalgar Square, London. New Phytologist.

[bib17] Gale A.S., Higgett J.M., Pälike H., Laurie E., Hailwood E.A., Hardenbol J. (2006). Correlation of Eocene–Oligocene marine and continental records: orbital cyclicity, magnetostratigraphy and sequence stratigraphy of the Solent Group, Isle of Wight, UK. Journal of the Geological Society of London.

[bib18] Gibbard P.L. (1985). The Pleistocene history of the Middle Thames Valley.

[bib19] Gibbard P.L. (1994). Pleistocene History of the Lower Thames Valley.

[bib20] Grün R.G., Schwarcz H.P. (2000). Revised open system U-series/ESR age calculations for teeth from Stratum C at the Hoxnian Interglacial type locality, England. Quaternary Science Reviews.

[bib21] Hill R.L. (1965). Hydrolysis of proteins. Advances in Protein Chemistry.

[bib22] Hollin J.T. (1977). Thames interglacial sites, Ipswichian sea levels and Antarctic ice surges. Boreas.

[bib23] Hughes S.A., 1987. The aminostratigraphy of British Quaternary non-marine deposits. Unpublished Ph.D. Thesis, University of Wales, Aberystwyth.

[bib24] Imbrie J., Shackleton N.J., Pisias N.G., Morley J.J., Prell W.L., Martinson D.G., Hays J.D., Macintyre A., Mix A.C., Berger A. (1984). The orbital theory of Pleistocene climate: Support from a revised chronology of the marine δ^18^O record. Milankovitch and Climate, Part 1.

[bib25] Kaufman D.S., Manley W.F. (1998). A new procedure for determining dl amino acid ratios in fossils using reverse phase liquid chromatography. Quaternary Science Reviews.

[bib26] Keen D.H. (1990). Significance of the record provided by Pleistocene fluvial deposits and their included molluscan faunas for palaeoenvironmental reconstruction and stratigraphy: case studies from the English Midlands. Palaeogeography, Palaeoclimatology, Palaeoecology.

[bib27] Kerney M.P. (1971). Interglacial deposits at Barnfield Pit, Swanscombe, and their molluscan fauna. Journal of the Geological Society, London.

[bib28] Lajoie K.R., Wehmiller J.F., Kennedy G.L., Hare P.E., Hoering T.C., King K. (1980). Inter- and intra-generic trends in apparent racemization kinetics of amino acids in Quaternary molluscs. Biogeochemistry of Amino Acids.

[bib29] Miller G.H., Mangerud J. (1985). Aminostratigraphy of European marine interglacial deposits. Quaternary Science Reviews.

[bib30] McCarroll D. (2002). Amino-acid geochronology and the British Pleistocene: secure stratigraphical framework or a case of circular reasoning?. Journal of Quaternary Science.

[bib31] Meijer T., Preece R.C. (2000). A review of the occurrence of *Corbicula* in the Pleistocene of North-West Europe. Geologie en Mijnbouw—Netherlands Journal of Geoscience.

[bib32] Miller G.H., Hare P.E. (1975). Use of amino acid reactions in some Arctic marine fossils as stratigraphic and chronologic indicators. Carnegie Institute of Washington Yearbook.

[bib33] Miller G.H., Hare P.E., Hare P.E., Hoering T.C., King K. (1980). Amino acid geochronology: integrity of the carbonate matrix and potential of molluscan fossils. Biogeochemistry of Amino Acids.

[bib34] Miller G.H., Hollin J.T., Andrews J.T. (1979). Aminostratigraphy of UK Pleistocene deposits. Nature.

[bib35] Miller G.H., Hart C.P., Roark E.B., Johnson B.J., Goodfriend G.A., Collins M.J., Fogel M.L., Macko S.A., Wehmiller J.F. (2000). Isoleucine epimerization in eggshells of the flightless Australian birds *Genyornis* and *Dromaius*. Perspectives in Amino Acid and Protein Geochemistry.

[bib36] Ovey, C.D., 1964. The Swanscombe Skull: a survey of research on a Pleistocene site. Occasional Paper No. 20, Royal Anthropological Institute.

[bib37] Penkman, K.E.H., 2005. Amino acid geochronology: a closed system approach to test and refine the UK model. Unpublished Ph.D. Thesis, University of Newcastle.

[bib38] Penkman, K.E.H., Kaufman, D.S., Maddy, D. and Collins, M.J., 2007. Closed-system behaviour of the intra-crystalline fraction of amino acids in mollusc shells. Quaternary Geochronology, doi:10.1016/j.quageo.2007.07.001. 10.1016/j.quageo.2007.07.001 PMC2727006 19684879

[bib39] Penkman K.E.H., Preece, R.C., Keen, D.H., Collins, M.J., accepted. Amino acid geochronology of the type Cromerian of West Runton, Norfolk, UK. Quaternary International. 10.1016/j.quaint.2010.06.020 PMC2991590 21217810

[bib40] Preece R.C., Bridgland D.R., Allen P., Haggart B.A. (1995). Mollusca from interglacial sediments at three critical sites in the Lower Thames. The Quaternary of the Lower Reaches of the Thames, Field guide.

[bib41] Preece R.C. (1999). Mollusca from Last Interglacial fluvial deposits of the River Thames at Trafalgar Square, London. Journal of Quaternary Science.

[bib42] Preece R.C., Penkman K.E.H. (2005). New faunal analyses and amino acid dating of the Lower Palaeolithic site at East Farm, Barnham, Suffolk. Proceedings of the Geologists’ Association.

[bib43] Roof S. (1997). Comparison of isoleucine epimerization and leaching potential in the molluskan genera *Astarte*, *Macoma*, and *Mya*. Geochimica et Cosmochimica Acta.

[bib44] Schreve D.C. (2001). Differentiation of the British late Middle Pleistocene interglacials: the evidence from mammalian biostratigraphy. Quaternary Science Reviews.

[bib45] Schreve D.C., Bridgland D.R., Allen P., Blackford J.J., Gleed-Owen C.P., Griffiths H.I., Keen D.H., White M.J. (2002). Sedimentology, palaeontology and archaeology of late Middle Pleistocene River Thames terrace deposits at Purfleet, Essex, UK. Quaternary Science Reviews.

[bib46] Schreve D.C., Harding P., White M.J., Bridgland D.R., Allen P., Clayton F., Keen D.H., Penkman K.E.H. (2006). A Levallois knapping site at West Thurrock, Lower Thames, UK: its Quaternary context, environment and age. Proceedings of the Prehistoric Society.

[bib57] Schreve, D.C., Keen, D.H., Limondin-Lozouet, N., Auguste, P., Santisteban, J.I., Ubilla, M., Matoshko, A., Bridgland, D.R., 2007. Progress in faunal biostratigraphy of Late Cenozoic fluvial sequences during IGCP 449. Quaternary Science Reviews 26, in press.

[bib47] Shotton F., Sutcliffe A.J., Bowen D.Q., Currant A.P., Coope G.R., Harmon R.S., Shackleton N.J., Stringer C., Turner, West R.G., Wymer J. (1983). Interglacials after the Hoxnian in Britain. Quaternary Newsletter.

[bib48] Sutcliffe, A.J., 1964. The mammalian fauna. In: Ovey, C.D. (Ed.), The Swanscombe Skull: a survey of research on a Pleistocene site. Occasional Paper No. 20, Royal Anthropological Institute, pp. 85–111.

[bib49] Sutcliffe A.J. (1975). A hazard in the interpretation of glacial–interglacial sequences. Quaternary Newsletter.

[bib50] Sutcliffe A.J., Bowen D.Q. (1973). Preliminary report on excavations at Minchin Hole. Pengelly Cave Studies Trust.

[bib51] Sykes G.A., Collins M.J., Walton D.I. (1995). The significance of a geochemically isolated intracrystalline fraction within biominerals. Organic Geochemistry.

[bib52] Towe K.M., Hare P.E., Hoering T.C., King K. (1980). Preserved organic ultrastructure: an unreliable indicator for Paleozoic amino acid biogeochemistry. Biogeochemistry of Amino Acids.

[bib53] Turner C., Kerney M.P. (1971). A note on the age of the freshwater beds of the Clacton Channel. Journal of the Geological Society of London.

[bib54] Wehmiller J.F., Stecher H.A., York L.L., Friedman I., Goodfriend G.A., Collins M.J., Fogel M.L., Macko S.A., Wehmiller J.F. (2000). The thermal environment of fossils: effective ground temperatures at aminostratigraphic sites on the US Atlantic Coastal Plain. Perspectives in Amino Acid and Protein Geochemistry.

[bib55] West R.G. (1969). Pollen analysis from interglacial deposits at Aveley and Grays, Essex. Proceedings of the Geologists’ Association.

[bib56] West R.G., Andrew R., Catt J.A., Hart C.P., Hollin J.T., Knudsen K.-L., Miller G.F., Penney D.N., Pettit M.E., Preece R.C., Switsur V.R., Whiteman C.A., Zhou L.P. (1999). Late and Middle Pleistocene deposits at Somersham, Cambridgeshire, UK: a model for reconstructing fluvial/estuarine depositional environments. Quaternary Science Reviews.

