# Supplementary material for: Testing the aminostratigraphy of fluvial archives: the evidence from intra-crystalline proteins within freshwater shells
Source: Quat Sci Rev. 2007 Nov;26(22-24):2958–69. doi: 10.1016/j.quascirev.2007.06.034 (PMC2727059; doi:10.1016/j.quascirev.2007.06.034)
Supplement: Supplementary file 2 — Online supplementary materials A [file mmc2.pdf]

Online supplement to

Testing the aminostratigraphy of fluvial archives: the evidence from intra-crystalline proteins within freshwater shells

by

K.E.H. Penkman, R.C. Preece, D.H. Keen, D. Maddy, D.C. Schreve and M.J. Collins

This online supplement includes data and detailed analysis results in support of the arguments in the main text. It includes six supplementary Figures, together with data and site information as a Microsoft Excel spreadsheet.

The spreadsheet, provided as a separate file, contains five worksheets, as follows:
Worksheet 1: Bleached amino acid data
Worksheet 2: Site information
Worksheet 3: Statistics: Thames sites
Worksheet 4: Statistics: Species effect
Worksheet 5: Unbleached amino acid data

Abbreviations and other notations used in the spreadsheet and supplementary Figures and their captions are explained in the main text. References cited in the spreadsheet, for details of study sites, are listed below.

References:

Ashton, N.M., Bowen, D.Q., Holman, J.A Hunt, C.O., Irving, B.G., Kemp, R.A., Lewis, S.G., McNabb, J., Parfitt, S., Seddon, M.B., 1994. Excavations at the Lower Palaeolithic site at East Farm, Barnham, Suffolk 1989-92. Journal of the Geological Society of London 151, 599-605.

Bowen, D.Q., 1999. A revised correlation of Quaternary deposits in the British Isles, Geological Society Special Report, No. 23

Bowen, D.Q., Hughes, S., Sykes, G.A., Miller, G.H., 1989. Land-sea correlations in the Pleistocene based on isoleucine epimerization in non-marine mollusks. Nature 340, 49-51

Bowen, D.Q., Sykes, G.A., Maddy, D., Bridgland, D.R., Lewis, S.G., 1995. Aminostratigraphy and amino acid geochronology of English lowland valleys: the Lower Thames in context. In: Bridgland, D.R., Allen, P., Haggart, B.A. (Eds), The Quaternary of the lower reaches of the Thames. Field guide, Quaternary Research Association, Durham, pp. 61-63.

Briant, R.M., 2002. Fluvial Responses to Rapid Climate Change in Eastern England during the last Glacial Period. Unpublished PhD thesis, University of Cambridge.

Briant, R.M., Coope, G.R., Preece, R.C., Gibbard, P.L., 2004. Evidence for Early Devensian (Weichselian) fluvial sedimentation: geochronological and palaeoenvironmental data from the Upper Pleistocene deposits at Deeping St James,

Lincolnshire, England. Quaternaire 15, 5-15
Bridgland, D.R., 1994. Quaternary of the Thames, Geological Conservation Review Series No 7.
Bridgland, D.R., Keen, D.H., Davey, N.D.W. 1991. The Pleistocene sequence in the Peterborough district: possible correlation with the deep-sea oxygen isotope record. In (Lewis, S. G., Whiteman, C. A. & Bridgland, D. R.; eds) Central East Anglia and the Fen Basin, Field Guide. Quaternary Research Association, London, 209–212
Bridgland, D.R., Field, M.H., Holmes, J.A., McNabb, J., Preece, R.C., Selby, I., Wymer, J.J., Boreham, S., Irving, B.G., Parfitt, S.A., Stuart, A.J., 1999. Middle Pleistocene interglacial Thames-Medway deposits at Clacton-on-Sea, England: Reconsideration of the biostratigraphical and environmental context of the type Clactonian Palaeolithic industry. Quaternary Science Reviews 18, 109-146.
Briggs D.J., Coope, G.R., Gilbertson, D.D., 1985. The chronology and environmental framework of early man in the Upper Thames valley: A new model. British Archaeological Report, British Series 137, 176
Buckingham, C.M., Roe, D.A., Scott, K., 1996. A preliminary report on the Stanton Harcourt Channel deposits (Oxfordshire, England): geological context, vertebrate remains and Palaeolithic stone artefacts. Journal of Quaternary Science 11, 397-415.
Currant, A., Jacobi, R., 2001. A formal mammalian biostratigraphy for the Late Pleistocene of Britain. Quaternary Science Reviews 20, 1707–1716.
Curry, D., Adams, C.G., Boulter, M.C., Dilley, F.C., Eames, F.E., Funnell, B.M., Wells, M.K., 1978 A correlation of Tertiary rocks in the British Isles, Geological Society of London Special Report, 12
Dixon, S.B., 1996. Molluscan signatures in the last Interglacial. Unpublished PhD thesis, Royal Holloway, University of London
Evans, J., Morse, E., Reid, C., Ridley, E.P., Ridley H.N., 1896. The relation of Palaeolithic man to the glacial epoch. Report of the British Association, Liverpool, pp. 400-416
Gale, A.S., Higgett, J.M., Pälike, H., Laurie, E., Hailwood, E.A., Hardenbol, J., 2006. Correlation of Eocene-Oligocene marine and continental records: orbital cyclicity, magnetostratigraphy and sequence stratigraphy of the Solent Group, Isle of Wight, UK. Journal of the Geological Society 163, 401-415. Gibbard, P.L., 1985. The Pleistocene history of the Middle Thames Valley. Cambridge University Press
Gibbard, P.L., 1985. The Pleistocene history of the Middle Thames Valley. Cambridge University Press.
Gibbard, P.L., 1994. Pleistocene history of the Lower Thames Valley. Cambridge University Press.
Green, C.P., Branch, N.P., Coope, G.R., Field, M.H., Keen, D.H., Wells, J.M., Schwenninger, J-L., Preece, R.C., Schreve, D.C., Canti, M.G., Gleed-Owen, C.P., 2006. Marine Isotope Stage 9 environments of fluvial deposits at Hackney, north London, UK. Quaternary Science Reviews 25, 89-113.
Grün, R., Schwarcz, H.P., 2000. Revised open system U-series/ESR age calculations for teeth from Stratum C at the Hoxnian Interglacial type locality, England. Quaternary Science Reviews 19, 1151-1154
Hollin, J.T., 1977. Thames interglacial sites, Ipswichian sea levels and Antarctic ice surges. Boreas 6, 33-52.

Holman, J.A., Claydon, J.D., 1990. A Late Pleistocene interglacial herpetofauna near Shropham, Norfolk. British Herpetological Society Bulletin 31, 31-35.
Holyoak D.T., Preece R.C., 1985. Late Pleistocene Interglacial Deposits at Tattershall, Lincolnshire. Philosophical Transactions of the Royal Society B 311, 193-236
Keen, D.H., 2001. Towards a late Middle Pleistocene non-marine molluscan biostratigraphy for the British Isles, Quaternary Science Reviews 20, 1657-1666
Keen, D.H., Bateman, M.D., Coope, G.R., Field, M,H,, Langford, H.E., Merry, J.S., Mighall, T.M., 1999. Sedimentology, palaeoecology and geochronology of Last Interglacial deposits from Deeping St. James, Lincolnshire, England. Journal of Quaternary Science 14 (5), 411-436.
Keen, D.H., Hardaker, T., Land, A.T.O., 2006. A Lower Palaeolithic industry from the Cromerian (MIS 13) Baginton Formation of Waverley Wood and Wood Farm Pits, Bubbenhall, Warwickshire, UK. Journal of Quaternary Science 21, 457-470
Kerney, M.P., 1971. Interglacial deposits at Barnfield Pit, Swanscombe, and their molluscan fauna. Journal of the Geological Society, London 127, 69-93.
Langford, H.E., Bateman, M.D., Penkman, K.E.H., Boreham, S., Briant, R.M., Coope, G.R., Keen, D.H. in press 2007. Age-estimate evidence for Middle-Late Pleistocene aggradation of River Nene 1st Terrace deposits at Whittlesey, eastern England. Proceedings of the Geologists' Association 118 (2).
Lewis, S.G., Maddy, D., Glenday, S., 2004. The Thames valley sediment conveyor: Fluvial system development over the last two interglacial-glacial cycles. Quaternaire 15, 17-28
Lewis, S.G., Maddy, D., Buckingham, C., Coope, G.R., Field, M.H., Keen, D.H., Pike, A.W.G., Roe, D.A., Scaife R.G., Scott, K., 2006. Pleistocene fluvial sediments, palaeontology and archaeology of the upper River Thames at Latton, Wiltshire, England. Journal of Quaternary Science 21 (2), 181-205.
Lightwing, J., 1982. Vertebrates from a New Site at Coston, Norfolk. Bulletin of the Geological Society of Norfolk 33, 73.
Maddy, D., Lewis, S.G., Scaife, R.G., Bowen, D.Q., Coope, G.R., Green, C.P., Hardaker, T., Keen, D.H., Rees-Jones, J., Parfitt, S., Scott, K., 1998. The Upper Pleistocene deposits at Cassington, near Oxford, England. Journal of Quaternary Science 13, 205-231.
Maddy, D., 1999. English Midlands: A correlation of Quaternary deposits in the British Isles. In: Bowen, D.Q. (Ed) Geological Society of London Special Report 23, 28-44
Preece, R.C., 1995. Mollusca from interglacial sediments at three critical sites in the Lower Thames. In: Bridgland, D.R., Allen, P., Haggart, B.A. (Eds), The Quaternary of the lower reaches of the Thames. Field guide, Quaternary Research Association, Durham, 53-62.
Preece, R.C., 1999. Mollusca from Last interglacial fluvial deposits of the River Thames at Trafalgar Square, London. Journal of Quaternary Science 14 (1), 77-89
Preece, R.C., Penkman, K.E.H., 2005. New faunal analyses and amino acid dating of the Lower Palaeolithic site at East Farm, Barnham, Suffolk. Proceedings of the Geologists' Association 116, 363-377.
Reid, C., 1882. The geology of the country around Cromer. Memoirs of the Geological Survey of England and Wales
Roe, H., 1995. The Cudmore Grove Channel Site. In: Bridgland, D.R., Allen, P., Haggart,

B.A. (Eds), The Quaternary of the lower reaches of the Thames. Field guide, Quaternary Research Association, Durham, 258-269.
Schreve, D.C. 2001a. Mammalian evidence from Middle Pleistocene fluvial sequences for complex environmental change at the oxygen isotope substage level. Quaternary International 79, 65-74
Schreve, D.C., 2001b. Differentiation of the British late Middle Pleistocene interglacials: the evidence from mammalian biostratigraphy. Quaternary Science Reviews 20, 1693-1705.
Schreve, D.C., Bridgland, D.R., Allen, P., Blackford, J.J., Gleed-Owen, C.P., Griffiths, H.I., Keen, D.H., White, M.J., 2002. Sedimentology, palaeontology and archaeology of late Middle Pleistocene River Thames terrace deposits at Purfleet, Essex, UK. Quaternary Science Reviews 21, 1423-1464.
Schreve, D.C., Harding, P., White, M.J., Bridgland, D.R., Allen, P., Clayton, F., Keen, D.H., Penkman, K.E.H., 2006. A Levallois knapping site at West Thurrock, Lower Thames, UK: its Quaternary context, environment and age. Proceedings of the Prehistoric Society 72, 21-52
Schwarcz, H.P., Grün, R., 1993. Electron Spin Resonance (ESR) dating of the Lower Industry, In Singer R, Gladfelter, B G and Wymer, J J (eds), The Lower Palaeolithic site at Hoxne, England, University of Chicago Press, 210-211
Scott, K., 2001. Late Middle Pleistocene Mammoths and Elephants of the Thames Valley, Oxfordshire. The World of Elephants - International Congress, Rome, 247-254
Shotton, F.W., 1983. Interglacials after the Hoxnian in Britain, In Billard, A, Conchon, O and Shotton, F W (eds) Quaternary glaciations in the northern hemisphere, UNESCO International Geological Correlation Programme, Project 73/1/24, Report 9, Paris, 109-115. Reproduced in Quaternary Newsletter 39, 20-5
Shotton, F.W., Keen, D.H., Coope, G.R., Currant, A.P., Gibbard, P.L., Aalto, M., Peglar, S.M., Robinson, J.E., 1993. The Middle Pleistocene deposits of Waverley Wood Pit, Warwickshire, England. Journal of Quaternary Science 8, 293-325.
Singer, R., Gladfelter, B.G., Wymer, J.J., 1993. The Lower Palaeolithic Site at Hoxne, England, University of Chicago Press, Chicago Sparks 1957
Stuart, A.J., 1976. The history of the mammal fauna during the Ipswichian/Last Interglacial in England. Philosophical Transactions of the Royal Society, London, B276, 221-250
Sutcliffe, A.J., 1975. A hazard in the interpretation of glacial-interglacial sequences. Quaternary Newsletter 17, 1-3
Walkling, A.P., 1996. Coleopteran records from the last interglacial-glacial transition. Unpublished PhD thesis, Royal Holloway, University of London
West, R.G., 1956. The Quaternary deposits at Hoxne, Suffolk. Philosophical Transactions of the Royal Society of London, B239, 265-356
West, R.G., 1969. Pollen analysis from interglacial deposits at Aveley and Grays, Essex. Proceedings of the Geologists' Association 80, 271-282
West, R.G., 1980. The pre-glacial Pleistocene of the Norfolk and Suffolk coasts. Cambridge University Press, Cambridge.

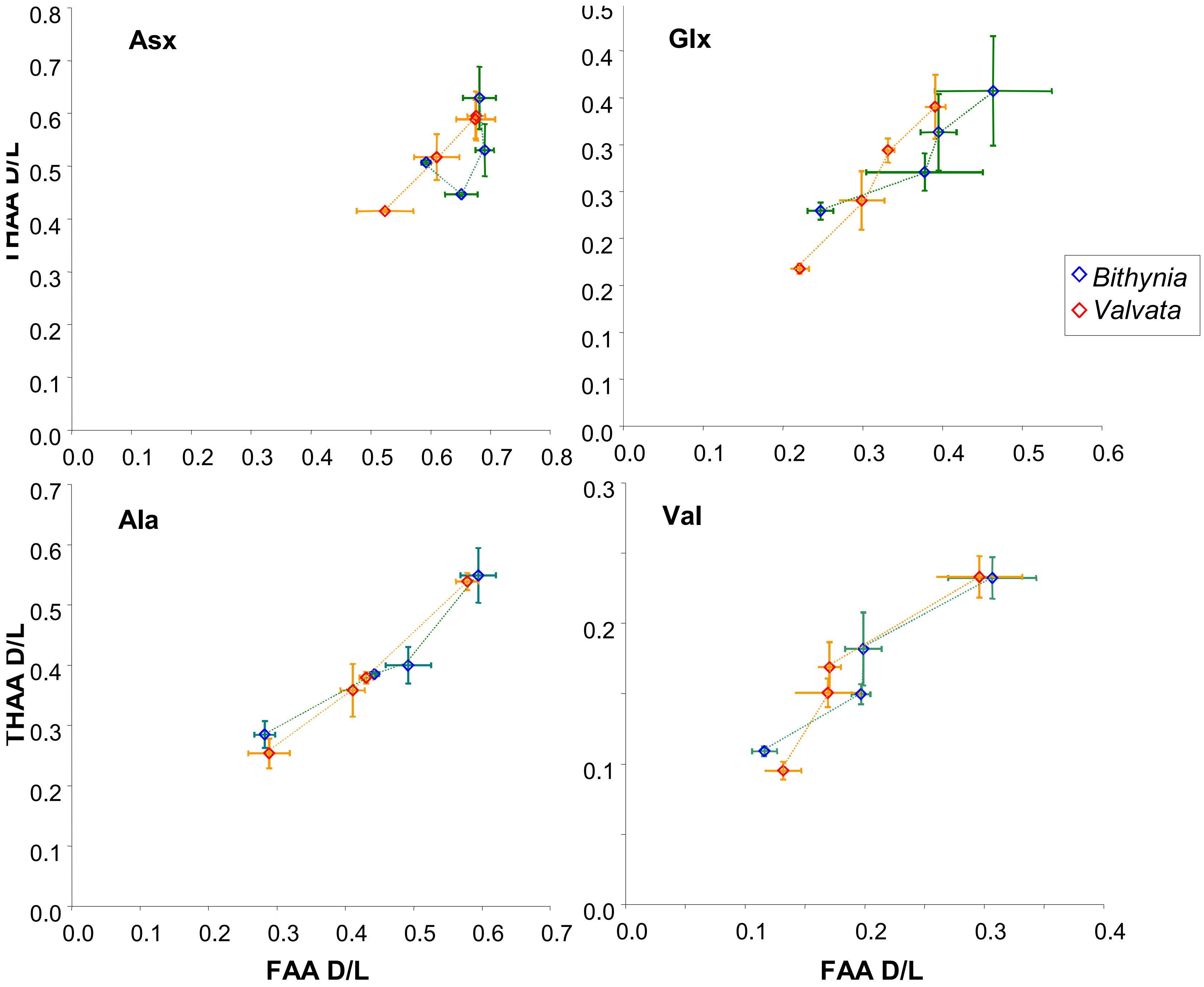

Asx
Glx
Ala
Val
Bithynia
Valvata
THAA D/L
FAA D/L
0.0
0.1
0.2
0.3
0.4
0.5
0.6
0.7
0.8

Figure S1 (previous page): FAA vs THAA for Asx, Glx, Ala and Val D/L in the bleached (intra-crystalline) fraction of *Bithynia tentaculata* and *Valvata piscinalis* shells from Trafalgar Square, Lion Pit / Aveley, Cudmore Grove and Clacton, with dotted lines joining the sites in order of age. Error bars represent one standard deviation about the mean for multiple samples. There are significant species differences in protein decomposition patterns, resulting in the need for species-specific aminostratigraphic frameworks.

Figure S2 (following page): FAA vs THAA for Asx, Glx, Ala and Val in the bleached (intra-crystalline) fraction of *Valvata piscinalis*. Error bars represent one standard deviation about the mean for the duplicate analyses.

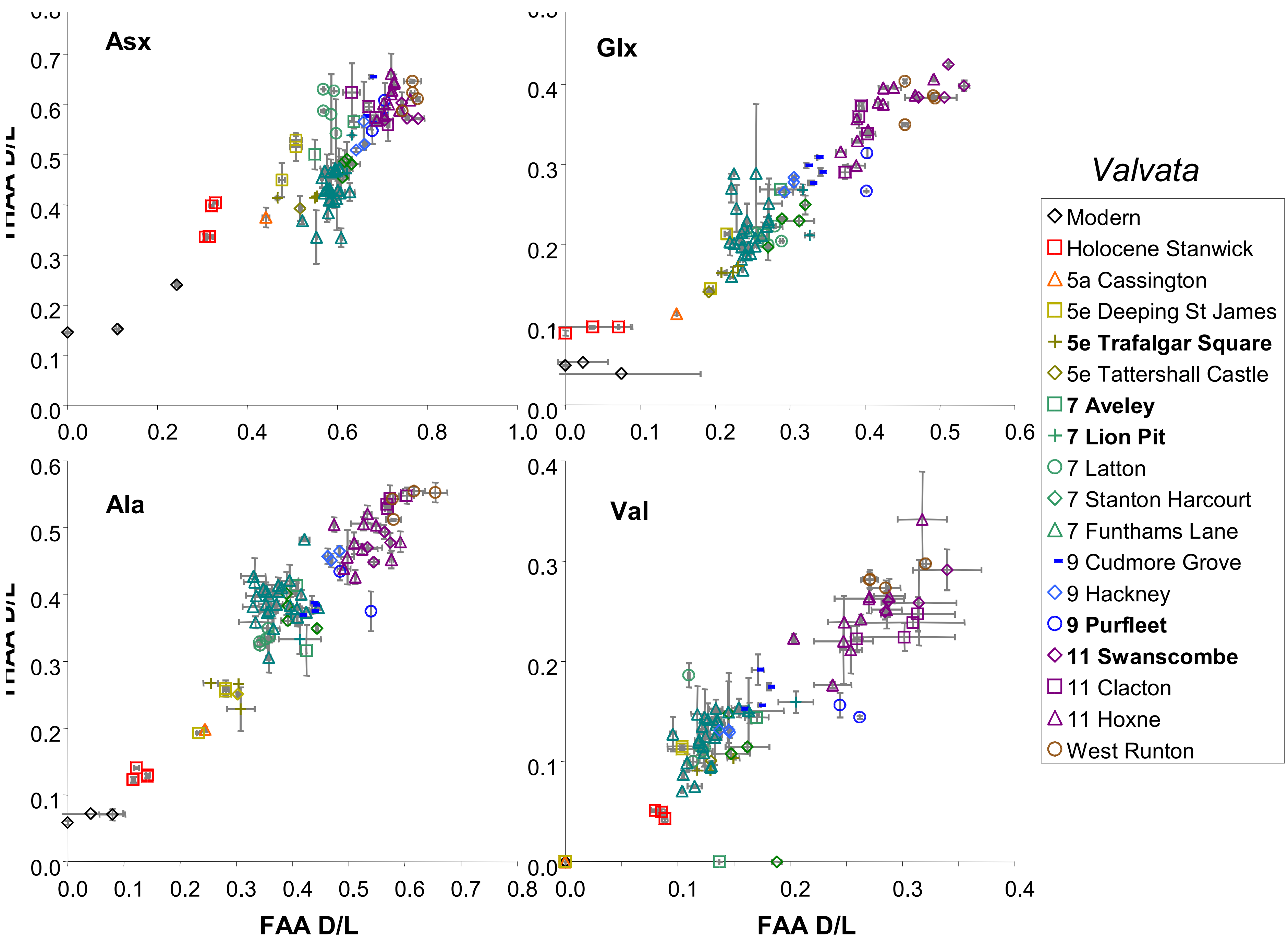

Asx
Glx
Ala
Val
THAA D/L
FAA D/L
Valvata
Modern
Holocene Stanwick
5a Cassington
5e Deeping St James
5e Trafalgar Square
5e Tattershall Castle
7 Aveley
7 Lion Pit
7 Latton
7 Stanton Harcourt
7 Funthams Lane
9 Cudmore Grove
9 Hackney
9 Purfleet
11 Swanscombe
11 Clacton
11 Hoxne
West Runton

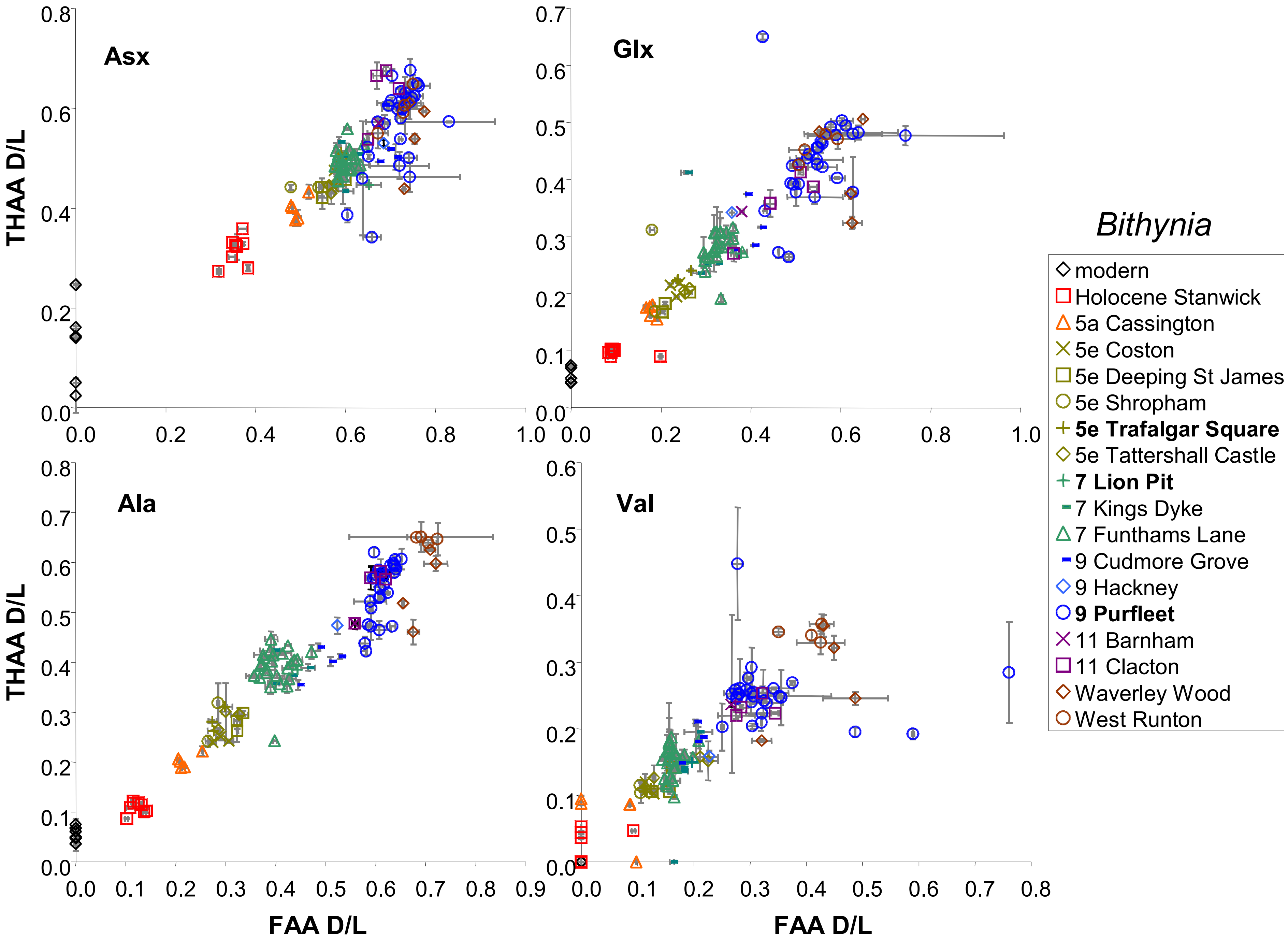

Asx
Glx
Ala
Val
THAA D/L
FAA D/L
Bithynia
modern
Holocene Stanwick
5a Cassington
5e Coston
5e Deeping St James
5e Shropham
5e Trafalgar Square
5e Tattershall Castle
7 Lion Pit
7 Kings Dyke
7 Funthams Lane
9 Cudmore Grove
9 Hackney
9 Purfleet
11 Barnham
11 Clacton
Waverley Wood
West Runton

Figure S3 (previous page).: FAA vs THAA for Asx, Glx, Ala and Val in the bleached (intra-crystalline) fraction of *Bithynia tentaculata* shell. Error bars represent one standard deviation about the mean for the duplicate analyses.

Figure S4 (following page): THAA vs FAA for Asx, Glx, Ala and Val in the unbleached (matrix and intra-crystalline) fraction of *Valvata piscinalis*. Error bars represent one standard deviation about the mean for the duplicate analyses.

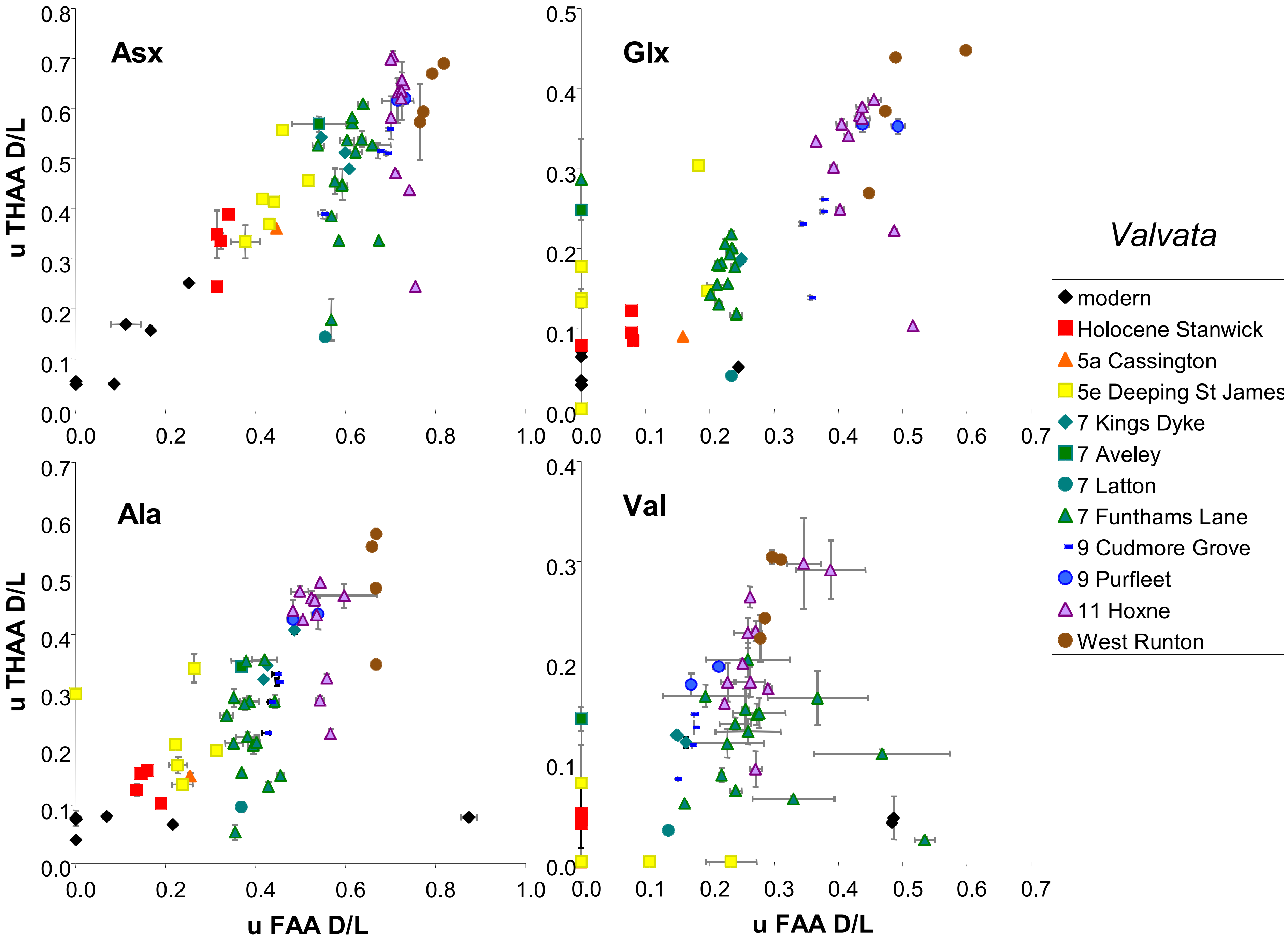
Asx
Glx
Ala
Val
u THAA D/L
u FAA D/L
Valvata
modern
Holocene Stanwick
5a Cassington
5e Deeping St James
7 Kings Dyke
7 Aveley
7 Latton
7 Funthams Lane
9 Cudmore Grove
9 Purfleet
11 Hoxne
West Runton

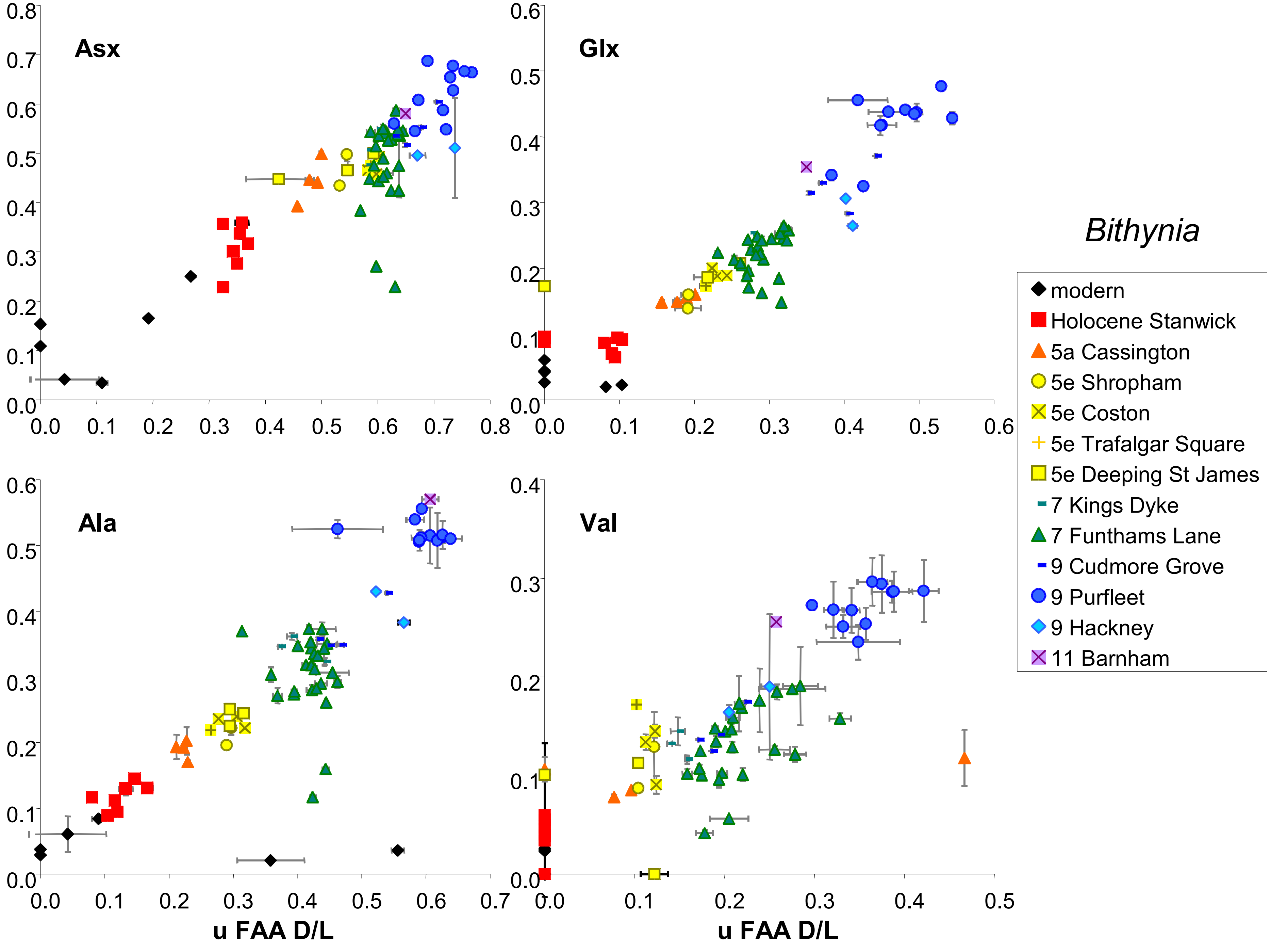
Asx
Glx
Ala
Val
u FAA D/L
u FAA D/L
Bithynia
modern
Holocene Stanwick
5a Cassington
5e Shropham
5e Coston
5e Trafalgar Square
5e Deeping St James
7 Kings Dyke
7 Funthams Lane
9 Cudmore Grove
9 Purfleet
9 Hackney
11 Barnham

Figure S5 (previous page): THAA vs FAA for Asx, Glx, Ala and Val in the unbleached (matrix and intra-crystalline) fraction of *Bithynia tentaculata* shell. Error bars represent one standard deviation about the mean for the duplicate analyses.

Figure S6 (following page).: THAA vs FAA for Asx, Glx, Ala and Val in the unbleached (matrix and intra-crystalline) fraction of *Corbicula fluminalis* shell.

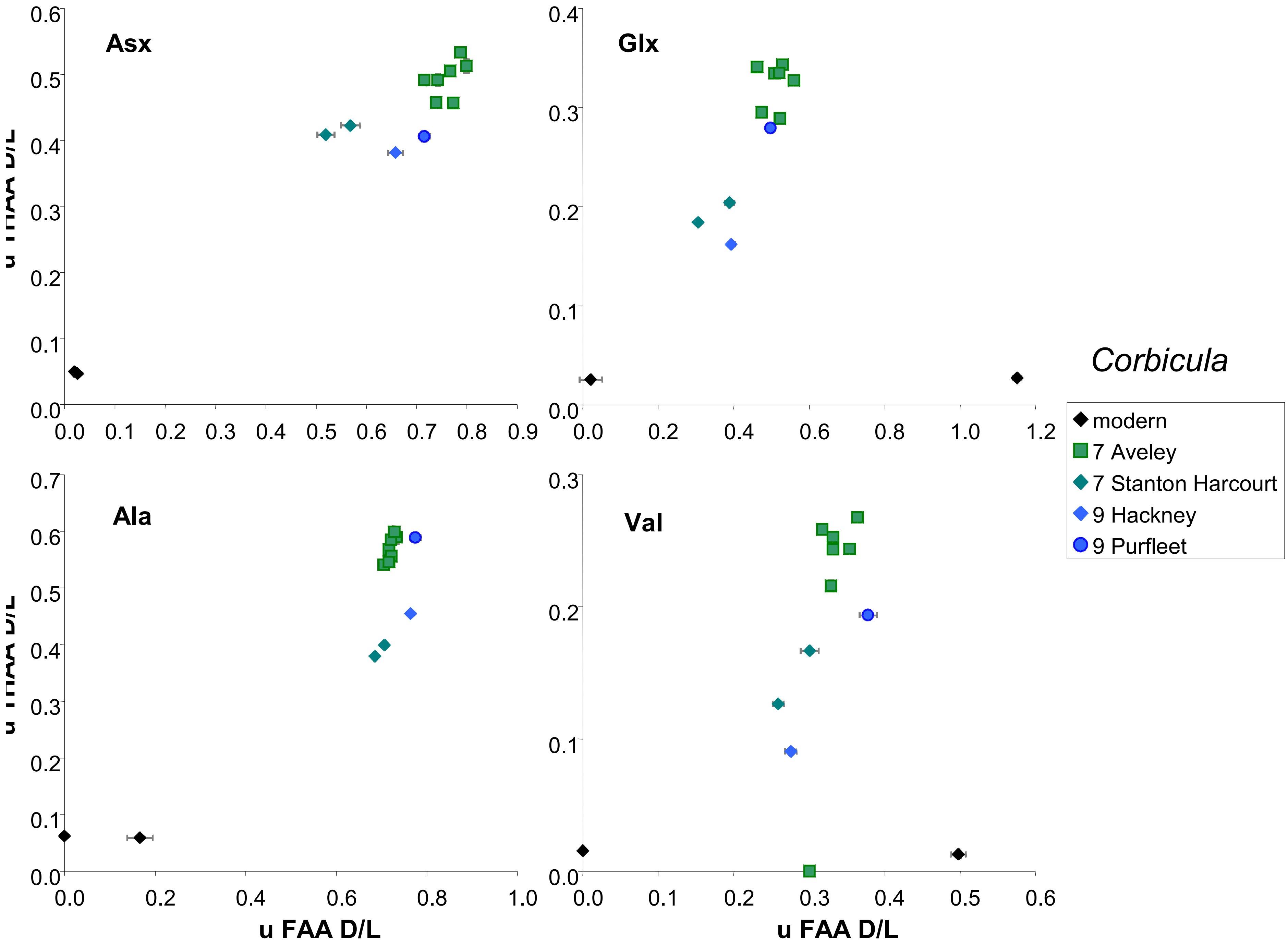
Asx
Glx
Ala
Val
u FAA D/L
u FAA D/L
Corbicula
modern
7 Aveley
7 Stanton Harcourt
9 Hackney
9 Purfleet
